# Emerging Understanding of the Mechanism of Action for Dimethyl Fumarate in the Treatment of Multiple Sclerosis

**DOI:** 10.3389/fneur.2018.00005

**Published:** 2018-01-23

**Authors:** Elizabeth A. Mills, Magdalena A. Ogrodnik, Andrew Plave, Yang Mao-Draayer

**Affiliations:** ^1^Department of Neurology, University of Michigan Medical School, Ann Arbor, MI, United States; ^2^Graduate Program in Immunology, Program in Biomedical Sciences, University of Michigan Medical School, Ann Arbor, MI, United States

**Keywords:** BG-12, lymphopenia, inflammation, neuroprotection, antioxidant

## Abstract

Dimethyl fumarate (DMF) is an effective treatment option for relapsing–remitting multiple sclerosis (MS), but its therapeutic mechanism of action has not been fully elucidated. A better understanding of its mechanism will allow for the development of assays to monitor its clinical efficacy and safety in patients, as well as guide the development of the next generation of therapies for MS. In order to build the foundation for determining its mechanism, we reviewed the manner in which DMF alters lymphocyte subsets in MS patients, its impact on clinical efficacy and safety, as well as its molecular effects in cellular and animal models. DMF decreases absolute lymphocyte counts, but does not affect all subsets uniformly. CD8^+^ T-cells are the most profoundly affected, but reduction also occurs in the CD4^+^ population, particularly within the pro-inflammatory T-helper Th1 and Th17 subsets, creating a bias toward more anti-inflammatory Th2 and regulatory subsets. Similarly, B-lymphocyte, myeloid, and natural killer populations are also shifted toward a more anti-inflammatory state. *In vitro* and animal models demonstrate a role for DMF within the central nervous system (CNS) in promoting neuronal survival in an Nrf2 pathway-dependent manner. However, the impact of DMF directly within the CNS of MS patients remains largely unknown.

## Introduction

Multiple sclerosis (MS) is an autoimmune disease that affects the central nervous system (CNS), characterized by an inappropriate inflammatory response to myelin associated autoantigens. MS is typically progressive in nature, resulting in demyelinating lesions and neurodegeneration. In patients with MS, the normal balance of pro-inflammatory and anti-inflammatory cells in the immune system is shifted toward inflammation in the CNS as well as in the periphery.

Many of the medications currently available for MS that have been shown to be effective in slowing the progression of the disease help restore the balance of immune cells toward a healthier state (1). This includes dimethyl fumarate (DMF), also known as BG-12, which has been used in the treatment of psoriasis since 1959 (2) and was FDA approved for the treatment of relapsing–remitting MS (RRMS) in 2013. The therapeutic mechanism of action for DMF is still unclear, but over the years, a better understanding of the biological pathways targeted by DMF has taken shape. In order to develop the next generation of therapies, it is critical to uncover these mechanisms and determine how DMF’s ability to shift the immune profile impacts both disease progression and the risk of adverse events, particularly opportunistic infections.

## Efficacy and Safety Profile

The clinical efficacy of DMF in MS has been investigated in two randomized placebo-controlled phase III clinical trials: DEFINE (3) and CONFIRM (4). In regard to annualized relapse rate (ARR), there was a 53% reduction of ARR in DEFINE and 44% reduction in the CONFIRM study with the 240 mg twice a day (BID) dosage compared to placebo, which ranks DMF higher than first-line injection treatment (3–5). The risk of confirmed disability progression sustained for 12 weeks as measured by the Expanded Disability Status Scale (EDSS) was reduced by 38% in DEFINE and 21% in CONFIRM studies in BID dosage. Both studies also demonstrated a reduction in the number of MRI lesions. “No evidence of disease activity” (NEDA) is a measure of therapeutic response, which takes into account relapses, sustained disability progression measured by EDSS, and MRI activity (6). NEDA has also been described as a potential indicator of brain atrophy and cognitive decline (7). The *post hoc* integrated analysis of the CONFIRM and DEFINE studies demonstrated a significant increase in clinical NEDA with a 38.9% relative reduction of disease activity over a 2-year period in comparison to placebo, as well as neuroradiological NEDA, with a 40.0% reduction in new or newly enlarging T2 hyperintense and gadolinium-enhanced lesions (7). A reduction in whole brain atrophy with DMF treatment was found in the DEFINE study and an independent pilot study (8, 9), but was not confirmed in the CONFIRM study (10). Furthermore, DMF was demonstrated to be a cost-effective treatment in RRMS (11).

Oral DMF was demonstrated to be a safe treatment for patients with RRMS during DEFINE and CONFIRM trials. The frequency of serious adverse events was comparable across all groups 18% (240 mg BID DMF), 16% (240 mg TID DMF), 21% placebo in DEFINE trial; 17% (240 mg BID DMF), 16% (240 mg TID), 17% glatiramer acetate, 22% placebo in CONFIRM trial with no opportunistic infections observed and malignancies accounting for less than 1% in all study groups (3, 4, 12). However, recent reports on safety show that there might be a correlation between DMF treatment and progressive multiple leukoencephalopathy (PML) (13).

Progressive multiple leukoencephalopathy is a potentially fatal condition that is caused by the lytic infection of glial cells by the JC polyomavirus, resulting in the loss of myelinating glial cells and progressive damage to the brain. To date, there have been five cases of PML in RRMS patients treated with DMF reported in both the literature and European pharmacovigilance databases (14–17), with another 14 cases described in patients treated with other formulas of DMF for psoriasis (17). Notably, 13 out of the 19 patients had grade 3 lymphopenia (17).

The development of PML has been widely studied in AIDS patients and is also known to be associated with hematological malignancies (18), and other immunomodulatory treatments, especially another drug approved for the treatment of RRMS, natalizumab. The average age of diagnosis is higher (58 years) for DMF-treated patients than for other groups of PML patients (40–45 years) (17). Notably, the risk of DMF-associated lymphopenia increases with age, with more severe lymphocyte count reduction (grade 2 or 3 lymphopenia) occurring in as many as 40% of DMF-treated MS patients above age 55 (19), which may explain the age bias in PML diagnosis. Meanwhile, there have been no reported cases of lymphopenia in the pediatric population (20). Overall, the incidence of grade 3 lymphopenia, defined as the absolute lymphocyte count less than 500/μl, is estimated to be between 2.2 and 9% (12, 21–23). Consequently, the Food and Drug Administration and European Medicines Agency set a guideline of absolute lymphocyte counts measuring less than 0.5 × 10^9^ for at least 6 months to consider halting use of DMF, as well as keeping patients under surveillance for PML (24). Despite the strong association, not all patients with DMF-associated PML experienced this type of prolonged overall lymphopenia (16, 25), suggesting that additional predictive metrics are still needed. A low CD4^+^/CD8^+^ ratio has been linked to greater risk for PML, with a CD4^+^ T-cell peripheral count less than 200 cells/μl used as an immunological predictor of PML in AIDS patients (26). However, DMF-associated PML is more commonly accompanied by very low counts of both CD4^+^ and CD8^+^ T-cells. In these patients, CD8^+^ T-cell lymphocytopenia may actually be a more reliable measure, since DMF treatment significantly affects this population, and a low CD8^+^ count is associated with a worse prognosis for PML patients (27).

## The Impact of DMF on the Peripheral Immune System

**Figure 1:**
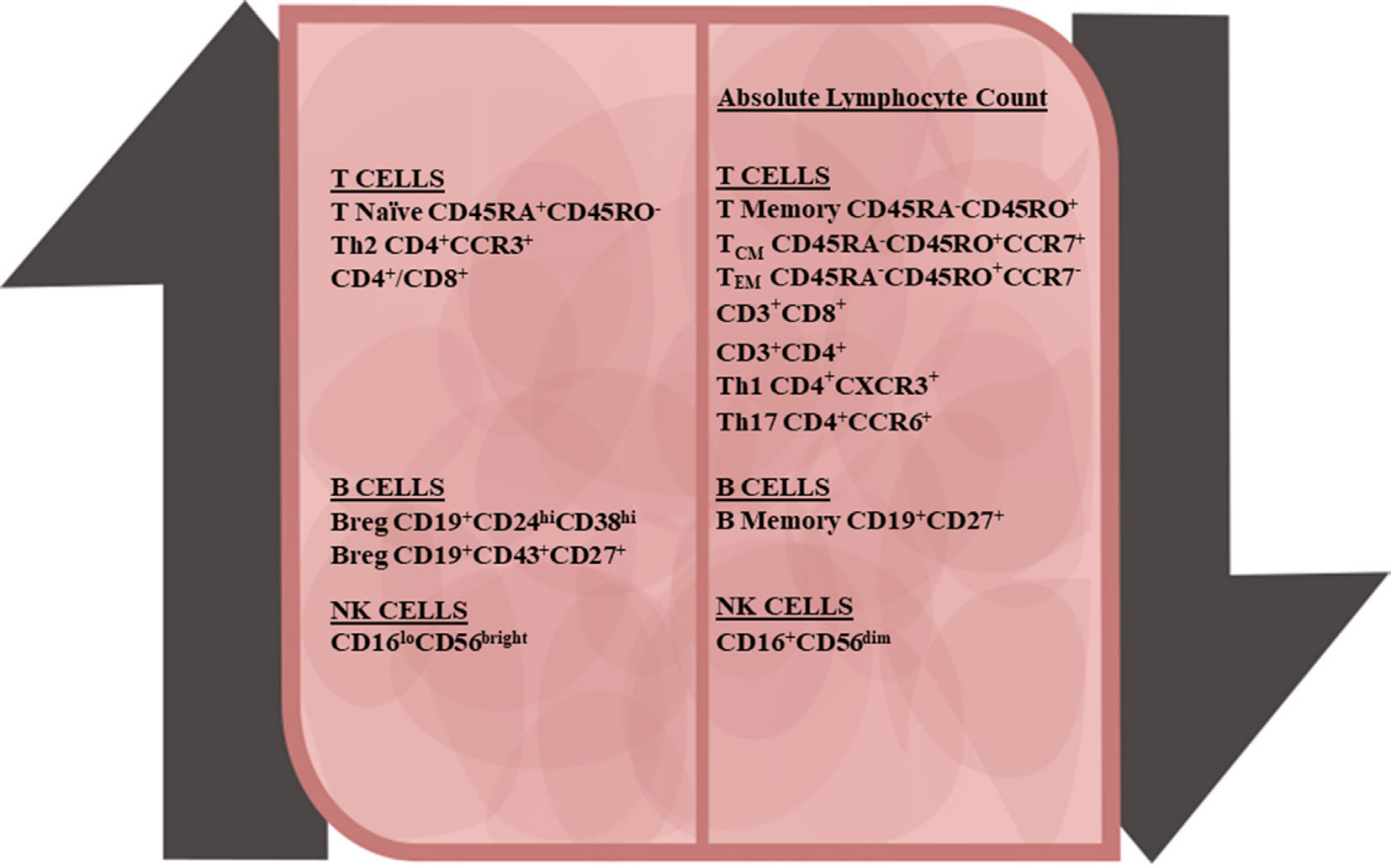
Peripheral immune cell changes due to dimethyl fumarate treatment in multiple sclerosis patients. Along with the decline in the absolute lymphocyte number, all major lymphocyte subsets also declined. T-cells demonstrated the following changes: increases in the naïve CD4^+^ and CD8^+^ T-cells and anti-inflammatory T-regulatory and Th2 subsets, and decreases in central memory T-cells, effector memory T-cells, and pro-inflammatory Th1 and Th17 T-cell subsets. In B-cells populations, there was an increase in transitional and B-regulatory subsets and decline in memory B-cells. With natural killer (NK) cells, CD56^bright^ NK cells were upregulated and CD56^dim^ NK cells were downregulated.

Treatment with DMF alters the profile of the immune system in terms of cell composition and inflammatory state (Figure 1). While the molecular mechanisms underlying these changes are still in the process of being elucidated, recent work from our group and others suggests that the shifted immune profile contributes to the therapeutic benefit of DMF and meanwhile likely increases the risk for PML. The majority of the proteins regulated by DMF treatment appear to have antioxidant and/or anti-inflammatory properties. For example, the DMF target, heme oxygenase-1 (HO-1), is an antioxidant, which also decreases expression of the pro-inflammatory cytokine IL-12 and stimulates T-regulatory (Treg) cells (28, 29). DMF was recently shown to be capable of modifying a variety of proteins involved in T-cell activation through its electrophilic activity (30). DMF was also found to reduce the production of nitric oxide synthase and the pro-inflammatory cytokines IL-1β, TNF-α, and IL-6 in cultured microglia (31), and to reduce NF-κB-mediated pro-inflammatory cytokine production in human peripheral blood mononuclear cells (32). Additionally, DMF is an agonist for the hydroxycarboxylic acid receptor (HCAR2) (33, 34). The binding of niacin, the endogenous ligand to HCAR2, was shown to reduce neuroinflammation, in part, through inhibition of NF-κB signaling (35), which likely contributes to the anti-inflammatory activity of DMF in MS patients. DMF also inhibits the production of pro-inflammatory cytokines by disrupting the association between toll-like receptor activation and downstream signaling pathways (36). Finally, DMF affects the survival of the immune cells themselves, and this effect on cell survival likely underlies the high incidence of lymphopenia associated with DMF. Although the peripheral absolute lymphocyte counts of MS patients treated with DMF decrease significantly (23, 37, 38), not all subpopulations of lymphocytes are affected equally. More work is needed to determine the full spectrum of immunological changes produced by DMF treatment in MS patients, but the studies performed so far suggest that pro-inflammatory subsets, particularly activated T-cells are disproportionately eliminated. A better understanding of the most critical subsets and continued monitoring of these subsets may improve our ability to identify poor responders, and improve treatment outcomes.

## Mechanism of Action Related to T Lymphocytes

The reduction in peripheral counts of T-cells following treatment with DMF was first noted in psoriasis patients (39), and only recently confirmed in MS patients by several groups, including our group (12, 22, 23, 38, 40). This loss of T-cells appears to be a direct dose-dependent effect of DMF, which occurs through the induction of apoptosis and decreased proliferation in these cells (40, 41). Although significant decreases in both CD4^+^ and CD8^+^ T-cell populations have been detected within 6 months of treatment, the extent of the loss was found to be greater for CD8^+^ cells, thereby producing an increase in the CD4^+^/CD8^+^ ratio (22, 40, 42). This is consistent with the finding that CD8^+^ T-cells are more susceptible to DMF-induced apoptosis *in vitro* (37). We noted a further decrease in CD8^+^ T-cells after 12 months of treatment, from a median of 0.40 × 10^6^/ml in untreated patients to a median of 0.17 × 10^6^/ml in patients with more than 18 months of treatment (40). Within memory fractions (CD45RA^−^CD45RO^+^), both central memory (Tcm) and effector memory (Tem) T cells were shown to be affected by DMF (40, 43). We found that CD4^+^ Tcm cells decrease with treatment durations of 4–6 months and greater than 18 months, while CD8^+^ Tcm cells slightly decrease initially, but eventually rebound (40). Meanwhile, both CD4^+^ and CD8^+^ Tem cells decrease significantly within 4–6 months of treatment, and then stabilize (40, 43). In contrast, the number of naïve (Tn) T-cells, CD3^+^CD4^+^, and CD3^+^CD4^−^ was found to be increased in DMF-treated patients for at least 18 months, compared to untreated patients (40, 43). These changes may underlie some of the therapeutic benefit of DMF, as decreased numbers of naïve cells and increased memory T-cells are thought to contribute to the development of RRMS (44, 45). Within these memory T-cell populations, the specific loss of activated and/or pro-inflammatory subsets is likely to be the most impactful. Indeed, we found that the percentage of activated CD69^+^ T-cells decreased with long-term DMF treatment (40). Furthermore, while we detected no change in the absolute number of terminally differentiated effector memory (Temra) T-cells, we saw a decrease in the percentage of CD69^+^ Temra following DMF treatment, indicative of reduced functional activation (40).

Perhaps more important than the absolute level of cell loss is the change in the relative distribution of the remaining subsets, particularly in relation to their inflammatory status. The changes in the composition of the peripheral blood of MS patients treated with DMF are consistent with its role in promoting a polarized shift toward a more anti-inflammatory state. The T-helper (Th) subsets CCXR3^+^ Th1 and CCR6^+^ Th17 secrete the pro-inflammatory cytokines IFN-γ and IL-17, which play a crucial role in MS pathogenesis (46, 47). Treatment with DMF decreases both the absolute number (43) and the proportion of these subsets relative to the total population of CD4^+^ T-cells (40, 42). Additionally, DMF reduces the number of CD161^+^ T-cells, which also contribute to IL-17 production (38). On the other hand, DMF increases the relative proportion of anti-inflammatory CCR3^+^ Th2 and Treg cells (40, 42), despite a decline in absolute cell number of Tregs (43). The increase in Th2 cells generally requires at least 6 months of treatment and is accompanied by an increase in production of IL-4 (40, 42). Meanwhile, the relative increase in Tregs, defined as CD4^+^ CD25^hi^CD127^lo^, likely stems from a decreased susceptibility of apoptosis following exposure to DMF, as compared to conventional CD4^+^CD25^−^ T-cells (37, 42). The increased Th2/Th1Th17 and Treg/Th1Th17 ratios are indicative of an anti-inflammatory shift and may be a measure of the clinical efficacy of DMF for MS patients.

## Mechanism of Action Related to Myeloid Cells

The anti-inflammatory shift in T-cells likely stems from DMF-induced changes in the maturation, availability, and antigen-presenting capacity of antigen-presenting cells. Treatment with DMF does not lead to significant changes in the absolute numbers of CD14^+^ monocytes or Lin1^−^HLADR^+^CD1c^+^BDCA4^−^BDCA2^−^ myeloid dendritic cells (DCs) in MS patients (40, 43). Instead, it is the polarization and function of the myeloid cells that is affected. Monocytes from DMF-treated RRMS patients have decreased expression of the pro-inflammatory micro-RNA miR-155, while DMF-treated monocyte-derived macrophages and microglia have decreased production of pro-inflammatory cytokines following lipopolysaccharide stimulation (48). In mice, DMF treatment does not affect conventional CD11c^hi^ DCs, but leads to decreased CD11b^+^ CD11c^−^ monocyte expression of co-stimulatory molecules CD80 and CD86 and induces a polarization bias in favor of the anti-inflammatory M2 phenotype (49). Furthermore, myeloid antigen-presenting cells from DMF-treated mice inhibit the differentiation of naïve T-cells into Th1 cells, and instead promote Th2 cell differentiation (49). This is consistent with *in vitro* assays indicating that DMF decreases the maturation and antigen-presenting capacity of DCs through the suppression of NF-κB and ERK1/2 pathways, ultimately resulting in decreased differentiation of Th1 and Th17 cells (50).

## Mechanism of Action Related to B Lymphocytes

Similar to its effect on T-lymphocytes, DMF treatment lowers the total levels of CD19^+^ B-lymphocytes in MS patients, and alters the profile of the remaining cells toward a more anti-inflammatory state (51–54). We were the first to show that the absolute number of CD27^+^ memory B-cells decreases significantly during the first 6 months of DMF treatment (51), which has subsequently been confirmed in other studies (53, 54). Reductions in memory B-cells also occur following treatment with other effective immunomodulatory therapies for MS (55), suggesting that the decrease in this cell population is relevant to clinical efficacy. We also found that the proportion of Breg subsets (CD24^high^CD38^high^, CD43^+^CD27^+^) are significantly increased following 12 months of treatment with DMF (51). CD24 ^high^ CD38^high^ B-cells are classified as transitional 2 marginal zone precursors and were found to increase in number at all examined time-points over 1 year (51). The greatest increase occurs in the CD43^+^CD27^+^ subset, which is known as an innate-like B1 IL-10-producing B-cell. The production of the anti-inflammatory cytokine IL-10 correlates with both subsets of Breg cells (CD24^high^CD38^high^ and CD43^+^CD27^+^) in MS patients treated with DMF in 12 months (51). Consistent with its anti-inflammatory bias, DMF treatment also results in a reduction in GM-CSF, IL-6, and TNF-α producing B-cells (53, 54), which are known for pro-inflammatory state enhancement (56, 57), and this decrease may be correlated with clinical efficacy (58). Although subsequent studies have failed to detect an effect of DMF on IL-10 (53, 54), the discrepancy likely stems from our finding that the increase of Breg was variable following short-term 4–6 months but consistently increased with long-term 12 months treatment. One of these studies grouped patients treated between 3 and 12 months (53), while the other did not examine patients treated for longer than 6 months (54). This suggests that changes in transitional B-cells and B-1 cells may occur later in the treatment course. Overall, the current literature shows that DMF shifts the B-lymphocyte profile away from pro-inflammatory memory subsets toward beneficial anti-inflammatory regulatory subsets. Larger studies with sequential longer term follow-up will be needed to confirm which subsets are the most clinically relevant.

## Mechanism of Action Related to Natural Killer (NK) Cells

Natural killer cells are involved in innate immunity and can be divided into immunomodulatory immature subsets that express high CD56 and low if any CD16 (CD56^bright^), and mature subsets with high cytotoxic activity that express CD16 and low CD56 (CD56^dim^). Various studies have found RRMS patients to exhibit a deficit in NK cell activity and shown a correlation between periods of low NK activity and lesion enhancement (59). This suggests that the ability of MS therapies to influence the activity of NK cells could impact their clinical efficacy. Indeed, therapies that have a long history of use for MS, including interferon-β and glatiramer acetate, have been shown to alter the balance of NK subsets toward more CD56^bright^ and/or enhance NK activity (59). Increases in CD56^bright^ cells have been shown to inhibit CD4^+^ and CD8^+^ IFN-γ-producing T-cell populations, thereby promoting an anti-inflammatory state (58). Similar to interferon-β, DMF treatment in MS patients upregulates CD56^bright^ cells and downregulates CD56^dim^ when compared to healthy controls (40, 43, 58). Additionally, one study suggests that the increase in CD56^bright^ cells is a potential indicator of drug efficacy (58). Our understanding of the contribution of NK cells to the efficacy of a particular MS therapy is still quite limited, but, as an increasing number of studies are also reporting changes to NK cells following treatment with newer more potent therapeutic agents (60), the role of NK cells is likely to garner further scrutiny.

## Mechanism of Action in CNS

**Figure 2:**
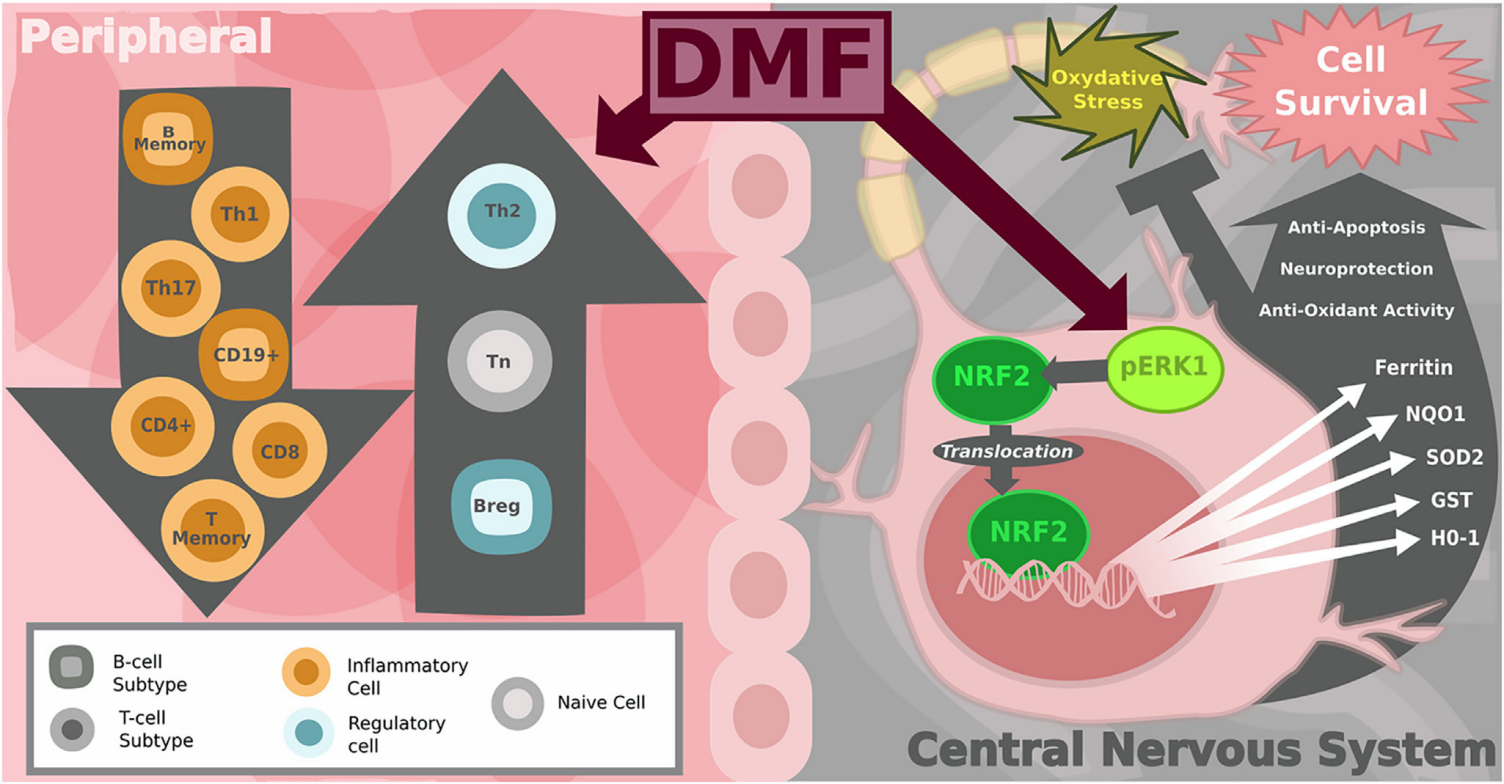
Diagram of peripheral immune and central nervous system (CNS) effects of dimethyl fumarate (DMF) in multiple sclerosis (MS). On the left: the shift in the balance toward anti-inflammatory immune cells in the peripheral blood. DMF-treated MS patients show a reduction in CD8^+^ and to lesser extent CD4^+^ T-cells as well as a decrease in the number of CD19^+^ B-cells. Subset analysis reveals that total B and T memory cells decline while the number of naïve T-cells increases. Pro-inflammatory T helper subsets Th1 and Th17 decrease, shifting the balance toward more anti-inflammatory Th2, T-regulatory and B-regulatory subsets. On the right: within the CNS, DMF and its metabolites are activators of the Nrf2-dependent intracellular pathway, which protects neurons from oxidative stress. Nrf2 translocates from the cytoplasm to the nucleus to increase transcription of genes encoding antioxidant enzymes, including: heme oxygenase-1 (HO-1), NAD(P)H quinone dehydrogenase 1 (NQO1), GSTP1 (others glutathione-S-transferase), superoxide dismutase-2 (SOD2), Sulfiredoxin-1 (SRXN1), ferritin heavy chain 1 (FTH1). Image copyright Caitlyn Fisher and Yang Mao-Draayer reprints with permission.

While the changes to the peripheral immune system are likely essential components to the efficacy of DMF in MS patients, efforts to determine the therapeutic mechanism of action that fail to account for its effects in the CNS are incomplete (see Figure 2). MS is a disease of neuroinflammation and neurodegeneration, thus the ability to influence neuronal or glial survival and the inflammatory state specifically within the CNS are critical components to consider for any MS therapy. DMF has long been known to protect cells facing oxidative stress through the activation of nuclear factor erythroid-2-related factor (Nrf2) from studies aimed at determining the mechanism related to its benefit for psoriasis patients (61). DMF-mediated induction of the Nrf2-ERK1/2 MAPK pathway leads to the increased expression of various antioxidant proteins such as HO-1, glutathione-S-transferase, superoxide dismutase, and quinone oxidoreductase-1 in various cell types (62–65). The upregulation of Nrf2 specifically within the CNS in the context of experimental allergic encephalomyelitis (EAE), a common rodent model of MS, was found to alleviate disease severity, thereby implicating a role for DMF within the cells of the CNS (64). Our work provides a potential mechanism for the CNS effects, as we showed that DMF treatment increases the frequency of neural stem/progenitor cells (NPCs) *in vitro* in rodents through an increase in self-renewal and protects NPCs and motor neurons from oxidative stress, leading to a decrease in stress-induced apoptosis and the production of reactive oxygen species (66).

It is unclear, however, what role DMF itself has on cells within the CNS in MS patients, since it is quickly hydrolyzed into monomethyl fumarate (MMF), which is able to cross the blood–brain barrier (BBB). MMF also appears to have neuroprotective properties, as it has been shown to reduce the severity of neuronal excitotoxicity induced by the local release of glutamate, which also plays a role in the pathogenesis of MS (67). These fumarates may also alter the distribution of immune cells in the CNS through modulation of transendothelial migration across the BBB. DMF induces the downregulation of the adhesive molecules: E-selectin, VCAM-1, and ICAM-1 (68, 69). DMF and MMF have been shown to reduce T-cell and macrophage infiltration in the spinal cord in an EAE mouse model (70), as well as inhibiting monocyte migration across inflamed human brain endothelial cells (71). However, at this point, no studies have examined the effect of DMF treatment on the distribution of lymphocyte subsets specifically within the CNS in MS patients. Since sampling from the CNS is not practical as a routine clinical measure, peripheral assays would be the most useful as diagnostic measures of response and efficacy, but a clinical analysis of CNS lymphocyte subset changes with DMF would be a valuable resource. This type of study would allow for comparisons with other MS therapies and provide the information necessary to improve our ability to effectively target pathogenic neuroinflammation in MS.

## Conclusion

In conclusion, the results of the studies described above indicate that DMF shifts the profile of peripheral lymphocyte subsets toward an anti-inflammatory state in MS patients. DMF treatment leads to a reduction in memory T-cells and a shifted balance toward less pro-inflammatory Th1/Th17 cells and more anti-inflammatory Th2 cells. While the effect of DMF on CNS lymphocyte subsets is currently unknown, recent studies indicate that DMF protects neural stem cells from oxidative damage *via* the Nrf2-ERK1/2 MAPK pathway. These findings are consistent with clinical improvement of patients treated with DMF.

## Author Contributions

EM, MO, AP, and YM-D contributed to the conceptual design and writing of this review.

## Conflict of Interest Statement

MO, AP, and EM have nothing to disclose. YM-D has served as a consultant and/or received grant support from: Acorda, Bayer Pharmaceutical, Biogen Idec, EMD Serono, Genzyme, Novartis, Questor, Genentech, and Teva Neuroscience.
